# Obstructive sleep apnea, cerebrovascular disease, and amyloid in older adults with Down syndrome across the Alzheimer’s continuum

**DOI:** 10.1093/sleepadvances/zpac013

**Published:** 2022-05-05

**Authors:** Patrick Lao, Molly E Zimmerman, Sigan L Hartley, José Gutierrez, David Keator, Kay C Igwe, Krystal K Laing, Dejania Cotton-Samuel, Mithra Sathishkumar, Fahmida Moni, Howard Andrews, Sharon Krinsky-McHale, Elizabeth Head, Joseph H Lee, Florence Lai, Michael A Yassa, H Diana Rosas, Wayne Silverman, Ira T Lott, Nicole Schupf, Adam M Brickman

**Affiliations:** Gertrude. H. Sergievsky Center, College of Physicians and Surgeons, Columbia University, New York, NY 10032, USA; Department of Neurology, College of Physicians and Surgeons, Columbia University, New York, NY 10032, USA; Taub Institute for Research on Alzheimer’s Disease and the Aging Brain, College of Physicians and Surgeons, Columbia University, New York, NY 10032, USA; Department of Psychology, Fordham University, Bronx, NY 10458, USA; Waisman Center, University of Wisconsin-Madison, Madison, WI, 53706, USA; Department of Neurology, College of Physicians and Surgeons, Columbia University, New York, NY 10032, USA; Department of Pathology & Laboratory Medicine, University of California, Irvine, Irvine, CA 92697, USA; Department of Neurology, College of Physicians and Surgeons, Columbia University, New York, NY 10032, USA; Department of Neurology, College of Physicians and Surgeons, Columbia University, New York, NY 10032, USA; Department of Neurology, College of Physicians and Surgeons, Columbia University, New York, NY 10032, USA; Department of Pathology & Laboratory Medicine, University of California, Irvine, Irvine, CA 92697, USA; Department of Neurology, College of Physicians and Surgeons, Columbia University, New York, NY 10032, USA; Gertrude. H. Sergievsky Center, College of Physicians and Surgeons, Columbia University, New York, NY 10032, USA; Department of Psychology, New York State Institute for Basic Research in Developmental Disabilities, Staten Island, NY 10314, USA; Gertrude. H. Sergievsky Center, College of Physicians and Surgeons, Columbia University, New York, NY 10032, USA; Department of Neurology, College of Physicians and Surgeons, Columbia University, New York, NY 10032, USA; Department of Psychology, New York State Institute for Basic Research in Developmental Disabilities, Staten Island, NY 10314, USA; Department of Pathology & Laboratory Medicine, University of California, Irvine, Irvine, CA 92697, USA; Gertrude. H. Sergievsky Center, College of Physicians and Surgeons, Columbia University, New York, NY 10032, USA; Department of Neurology, College of Physicians and Surgeons, Columbia University, New York, NY 10032, USA; Department of Epidemiology, Mailman School of Public Health, Columbia University, New York, NY 10032, USA; Department of Neurology, Massachusetts General Hospital, Harvard University, Boston, MA 02114, USA; Department of Pathology & Laboratory Medicine, University of California, Irvine, Irvine, CA 92697, USA; Department of Neurology, Massachusetts General Hospital, Harvard University, Boston, MA 02114, USA; Department of Radiology, Athinoula Martinos Center, Massachusetts General Hospital, Harvard University, Charlestown, MA 02129, USA; Department of Pediatrics, University of California, Irvine, Irvine, CA 92697, USA; Department of Pediatrics, University of California, Irvine, Irvine, CA 92697, USA; Gertrude. H. Sergievsky Center, College of Physicians and Surgeons, Columbia University, New York, NY 10032, USA; Department of Neurology, College of Physicians and Surgeons, Columbia University, New York, NY 10032, USA; Department of Psychiatry, College of Physicians and Surgeons, Columbia University, New York, NY 10032, USA; Gertrude. H. Sergievsky Center, College of Physicians and Surgeons, Columbia University, New York, NY 10032, USA; Department of Neurology, College of Physicians and Surgeons, Columbia University, New York, NY 10032, USA; Taub Institute for Research on Alzheimer’s Disease and the Aging Brain, College of Physicians and Surgeons, Columbia University, New York, NY 10032, USA

**Keywords:** Down syndrome, Alzheimer’s disease, Cerebrovascular disease, Obstructive sleep apnea

## Abstract

We determined the extent to which obstructive sleep apnea (OSA) is associated with increased cerebrovascular disease and amyloid burden, and the relation of the two processes across clinical Alzheimer’s disease (AD) diagnostic groups in adults with Down syndrome (DS). Adults with DS from the Biomarkers of Alzheimer’s Disease in Down Syndrome (ADDS) study were included given available research MRI (*n* = 116; 50 ± 8 years; 42% women) and amyloid PET scans (*n* = 71; 50 ± 7 years; 39% women) at the time of analysis. Participants were characterized as cognitively stable (CS; 64%), with mild cognitive impairment-DS (MCI-DS; 23%), with possible AD dementia (5%), or with definite AD dementia (8%). OSA was determined via medical records and interviews. Models tested the effect of OSA on MRI-derived cerebrovascular biomarkers and PET-derived amyloid burden, and the moderating effect of OSA and AD diagnosis on biomarkers. OSA was reported in 39% of participants, which did not differ by clinical AD diagnostic group. OSA was not associated with cerebrovascular biomarkers but was associated with greater cortical amyloid burden. White matter hyperintensity (WMH) volume (primarily in the parietal lobe), enlarged perivascular spaces, and cortical and striatal amyloid burden were greater across clinical AD diagnostic groups (CS<MCI-DS<possible AD<definite AD). OSA increased the differences in WMH volumes across clinical AD diagnostic groups, primarily in the frontal and temporal lobes. Adults with DS and OSA had greater amyloid burden and greater cerebrovascular disease with AD. Importantly, OSA may be a modifiable risk factor that can be targeted for intervention in this population at risk for AD.

## Graphical Abstract

**Figure:**
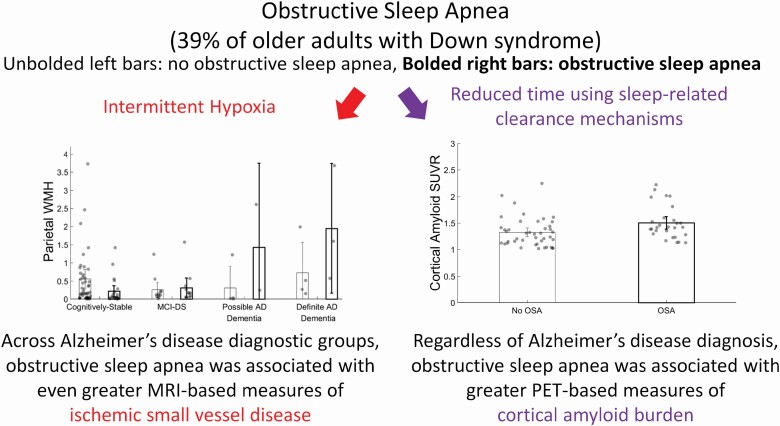

**Statement of Significance**

Adults with Down syndrome (DS) have a triplicate copy of chromosome 21 and overproduce amyloid precursor protein, increasing risk for Alzheimer’s disease (AD). Alzheimer’s biomarkers include amyloid and cerebrovascular disease, which increase across the AD continuum in adults with DS. Obstructive sleep apnea (OSA), characterized by intermittent hypoxia and reduced time in sleep states when clearance mechanisms are operative, was reported in 39% of older adults with DS (>40 years). OSA was not associated with greater cerebrovascular disease, but interacted with AD diagnosis, suggesting that OSA treatment may improve cerebrovascular outcomes during AD progression in this population. Adults with DS and OSA had a greater amyloid burden compared to adults with DS without OSA, regardless of AD diagnosis.

## Introduction

Adults with Down syndrome (DS) have a triplicated copy of chromosome 21 with overexpression of the amyloid precursor protein (APP) gene as the main contributor to the high risk of Alzheimer’s disease (AD) in this population [1,2]. Elevated amyloid burden emerges in nearly all adults with DS by the age of 40 years [3] and clinical impairment is present in nearly all individuals in their 60s [4]. However, despite this consistent overproduction of APP, there is a wide range in the age of onset of dementia in adults with DS and growing evidence that other pathogenic processes may contribute to AD in this population. One potential factor is sleep-related disturbances, which are common in adults with DS. Importantly, sleep-related disturbances may be modifiable and thus may offer a pathway to alter the timing of AD onset in the DS population.

Obstructive sleep apnea (OSA) is one sleep disorder characterized by repeated blockage or narrowing of the upper airway during sleep that results in sleep fragmentation and dips in blood oxygenation, or intermittent hypoxia. Based on the reported medical diagnosis of OSA, estimates in adults with DS range from 13 to 49% [5–8], compared with 2 to 6% in adults without DS [9,10]. However, studies of direct measures of OSA (e.g. polysomnographs) suggest that OSA may occur in more than 78% of adults with DS [11,12], compared with 9–38% in adults without DS [13]. The high prevalence of OSA in adults with DS is largely thought to be related to musculature differences in the upper airway [14], although other factors including medications or degeneration of brain areas that modulate sleep could also play a role. OSA in older adults without DS is associated with an earlier age of onset for mild cognitive impairment (MCI) and AD [15], and with a higher risk for incident AD dementia [16]. In younger and middle-aged adults without DS, OSA is associated with memory and attention impairments, potentially due to sleep fragmentation [17] and excessive daytime sleepiness [18], as well as with executive function impairments, potentially due to the severity of hypoxemia [19]. In line with these findings, MRI studies demonstrated that OSA is associated with lower hippocampal volume, parietal lobe volume, and frontal lobe volume [20], as well as with lower white matter microstructure in similar regions [21]. PET studies in late-onset AD also showed that OSA is associated with greater amyloid [22,23] and tau [24] burden, as well as accelerated amyloid [25,26] and tau [26] deposition rates; although findings have been mixed [27] and differ based on the disease stage (e.g. cognitively normal vs MCI vs AD) and the sample (e.g. clinical vs sleep clinic vs community-based). OSA may contribute to AD risk through intermittent hypoxia and poorer clearance of amyloid during fragmented sleep, resulting in higher burden of cerebrovascular disease and AD pathology, respectively. Additionally, there may be a link between ischemic small vessel disease and tau phosphorylation [28], suggesting that OSA may also contribute to tau pathology through small vessel disease. In adults with DS, OSA is associated with lower verbal intellectual functioning and cognitive flexibility [29]. While OSA is more common in adults with DS, the mechanisms by which it affects cognition and AD are thought to be similar to those in adults without DS [14].

In adults with DS, despite the low prevalence of traditional vascular risk factors, such as hypertension, there is MRI evidence of greater cerebrovascular disease from those who are cognitively-stable (CS) to mild cognitive impairment (MCI-DS), possible AD, and definite AD [30]. Adults with DS demonstrated monotonically greater white matter hyperintensity (WMH) volume across clinical AD diagnostic groups (CS < MCI-DS < possible AD < definite AD) that was most pronounced in the parietal lobe, above and beyond what would be expected with age. Enlarged perivascular spaces (PVS) and infarcts were greater across clinical AD diagnostic groups, but did not differ after age adjustment, suggesting a weaker link to AD. Similar findings have been observed in autosomal dominant AD in which individuals were too young to have considerable vascular risk factors, but showed an early increase in parietal and occipital WMH volumes prior to dementia onset [31]. In late-onset AD, parietal WMH are higher compared with individuals without dementia and thought to be a better predictor of incident AD compared with hippocampal volume [32]. Therefore, cerebrovascular disease may be involved in the pathogenesis or symptom expression of AD, and not simply a manifestation of vascular risk factors.

Previous work in adults with DS has laid the groundwork for this study by characterizing the development of cerebrovascular disease and amyloid burden across clinical AD diagnostic groups. The current analyses extend this work by investigating OSA as one potential mechanism by which cerebrovascular disease and amyloid burden may contribute to the development and progression of clinical AD. There were two goals in the present study. First, image-based biomarkers of cerebrovascular disease (including WMH, enlarged PVS, infarcts, and microbleeds) and amyloid burden were compared in individuals with DS with and without OSA. We hypothesized that those with OSA would have a higher burden of cerebrovascular disease and amyloid burden than those without OSA due to transient ischemia and reduced time for sleep-based clearance mechanisms, respectively. Second, biomarker increases across clinical AD diagnostic groups were compared in individuals with DS with and without OSA. We hypothesized that cerebrovascular disease and the amyloid burden would increase across clinical AD diagnostic groups to a greater extent in those with OSA compared to those without OSA.

## Methods

### Participants

Participants were enrolled in the Biomarkers of Alzheimer’s Disease in Adults with Down Syndrome (ADDS; U01 AG051412) study. The primary goal of ADDS is to characterize the development of clinical AD among adults with DS using clinical, genetic, and biomarker (e.g. cerebrospinal fluid, blood, and neuroimaging) data. To date, ADDS has enrolled over 200 individuals 40 years old and above at Columbia University/New York State Institute for Basic Research in Developmental Disabilities, Massachusetts General Hospital/Harvard Medical School, and University of California-Irvine. The study was approved by the Institutional Review Boards at participating institutions and written informed consent was obtained from participants and/or their legal guardian or legally authorized representative. Furthermore, we received assent from every participant before every procedure.

### Obstructive sleep apnea

Analyses included participants, reported on in our previous study [30], with a clinical AD consensus diagnosis, MRI sequences required for cerebrovascular disease quantification, and amyloid PET, with the additional restriction that information about OSA be available. The presence (vs. absence) of OSA was reported by participants or their informants as part of their health history or obtained from medical/health records. Furthermore information about the duration of OSA (not present, lifelong condition, past 5 years, and past year) and the treatment of OSA (not present, inactive, active treated, and active untreated) were also collected as part of the National Task Group Early Detection Screen Test [33] and the health history, respectively. Information about the type of OSA treatment were not available for this study. Data including body mass index (BMI), apolipoprotein (APOE) allele, medications, and medical history of vascular risk factors were also collected.

### Clinical assessment

Full descriptions of the clinical assessments, as well as MRI and PET scanning procedures are provided elsewhere [30]. Briefly, a consensus panel including clinicians with expertise in DS-specific assessments adjudicated one of the four clinical AD diagnostic groups following standardized procedures (i.e. using neuropsychological testing in AD-relevant domains, clinical chart reviews, and interviews with knowledgeable informants, while considering health history, functional and vocational abilities, and neuropsychiatric symptoms), blinded to neuroimaging or other AD biomarkers. In cognitively-stable (CS) individuals there was no evidence of clinically significant cognitive decline beyond preclinical intellectual functioning and age. Mild cognitive impairment-Down syndrome (MCI-DS) indicated evidence of cognitive decline over time beyond preclinical intellectual functioning and age, but insufficient to suggest dementia. Possible AD dementia indicated evidence of substantial decline of breadth and severity greater than MCI-DS, but declines over time were judged to need furthermore evidence of progression. Definite AD dementia indicated clear evidence of substantial cognitive and functional decline with a high degree of confidence in the dementia rating. For MCI-DS, possible AD dementia, and definite AD dementia, findings were reviewed to establish AD as the primary etiological diagnosis. Three participants in the neuroimaging sample (2%) were excluded based on complications or concerns unrelated to neurodegenerative disorders (e.g. severe sensory loss, new psychiatric diagnosis).

### MRI imaging

Multisite MRI scans were performed on a Siemens Prisma 3T at Columbia University (*n* = 29) and MGH (*n* = 55) or a Philips Achieva 3T at UC-Irvine (*n* = 57) using Alzheimer’s Disease Neuroimaging Initiative protocols (T1-weighted scan: repetition time (TR)/echo time (TE)/inversion time (TI): 2300/2.96/900ms; voxel size: 1x1x1mm^3^; T2-weighted fluid attenuated inversion recovery (FLAIR) scan (TR/TE/TI: 5000/386/1800ms; voxel size: 0.4x0.4x0.9mm^3^). White matter hyperintensity volume (WMH), which likely represents the extent of ischemic small vessel disease, was quantified from T2-weighted FLAIR scans; briefly, scans were skull stripped, intensity normalized, high pass filtered at the mode, log-transformed, fit with a half-Gaussian mixture model, labeled as hyperintense if voxels within the higher full Gaussian distribution formed a contiguous cluster atleast 5 voxels, and manually edited as necessary [34]. Total WMH volume, as well as lobar WMH volumes, were estimated. Enlarged perivascular spaces (PVS), which likely represent the integrity of vascular clearance mechanisms of toxins from the brain, were visually counted on T1 scans as hypointensities across 13 brain regions and rated from 0 to 2 based on FLAIR characteristics (hyperintense ring). We investigated a global score ranging from 0 (no enlarged PVS in any region) to 26 (most severe enlarged PVS in each region) [35]. Infarcts, which represent large vessel disease, were visually counted on FLAIR scans (discrete hypointense lesions > 5mm with a partial or complete hyperintense ring), and confirmed on T1 scans (hypointense areas) by an expert rater. We investigated infarcts globally as a dichotomous variable (none vs at least one). Microbleeds, which likely result from cerebral amyloid angiopathy (CAA), were rated by visual inspection, as hypointense round or ovoid lesions on GRE (*n* = 28) or SWI (*n* = 47), surrounded at least half way by parenchyma with a “blooming” effect and no hyperintensity on accompanying T1-weighted or FLAIR scans to distinguish them from iron or calcium deposits, bone, or vessel flow voids [36–38]. Microbleeds were scored globally as present or not.

### PET imaging

A smaller subset of participants underwent amyloid PET ([^18^F]Florbetapir/AV45) on a Siemens Biograph 64 at Columbia University (voxel size=1x1x2mm^3^, reconstruction = OSEM3D+TOF, *n* = 10), on a Siemens Biograph mMR at MGH (voxel size = 2.1x2.1x2.0mm^3^, reconstruction = OP-OSEM, *n* = 31), or a Siemens high resolution research tomograph (HRRT) at UC-Irvine (voxel size =1.2x1.2x1.2mm^3^, reconstruction = OP-OSEM3D, *n* = 30), using the ADNI protocol (4x5 min frames; 50–70 min post-injection) [39], and correcting for attenuation, radioactive decay, detector normalization, randoms, and scatter. Using FreeSurfer (v 6.0) defined regions [40], standard uptake value ratios (SUVRs), with and without partial volume correction (PVC; PETSurfer [41]), were calculated in PET native space using the cerebellar gray matter as reference region for the cortex (weighted average of anterior cingulate, frontal cortex, parietal cortex, precuneus, and temporal cortex, according to Thal phasing of amyloid deposition in late-onset AD [42]) and the striatum alone due to its early amyloid accumulation in adults with DS [43,44].

### Statistical approach

In the ADDS participants with OSA information, regardless of neuroimaging (*n* = 173), the frequency of OSA was not different between those with (39%) and without (30%) MRI data (χ ^2^(1) = 1.3, *p* = 0.25) or those with (39%) and without (33%) amyloid PET data (χ ^2^(1) = 0.7, *p* = 0.41), suggesting that those with OSA were not more likely to be excluded from imaging. Statistical analyses were performed for each biomarker separately due to different sample sizes for each imaging modality (i.e. not completing a particular sequence, poor scan quality, motion, etc; WMH, *n* = 102; enlarged PVS, *n* = 111; microbleeds, *n* = 75; infarcts, *n* = 111; amyloid SUVR, *n* = 71). Missingness for each biomarker was not associated with age, sex, the presence of an APOE-ε4 allele, clinical AD diagnosis, or OSA, except that microbleed reads were more likely to be missing with increasing age (Odds ratio [95% CI], *p*-value; 3.8 [1.1, 6.5], *p* = 0.006). Differences by OSA in demographic data (including age, sex, APOE-ε4, and clinical AD diagnosis) and neuroimaging biomarkers of cerebrovascular disease and amyloid were tested with t-tests, chi-squared tests, general linear models (e.g. linear regression), or generalized linear models (e.g. logistic regressions), depending on the outcome variable type and the inclusion of an imaging site covariate for neuroimaging biomarkers. We also report available information on OSA duration and treatment, but did not consider these factors in analytic models due to small cell sizes.

Linear regression models were used to test for monotonically greater biomarker levels (i.e. WMH volume, PVS scores, amyloid SUVR) across clinical AD diagnostic groups (CS < MCI-DS < possible AD < definite AD) including an interaction term with OSA and adjustment for age and imaging site; similarly, logistic regression models were used to test for a monotonically greater likelihood of infarcts or microbleeds across clinical AD diagnostic groups including an interaction term with OSA and adjustment for age and imaging site (results presented as odds ratios). We tested monotonic trends across diagnostic groups because adults with DS, at any given time, are on a continuum of AD progression and severity. All statistical tests were performed in IBM SPSS Statistics v26, and *post hoc* analyses involving lobar WMH associations were corrected for multiple comparisons using the False Discovery Rate (FDR).

## Results

Overall, 45 (39%) participants had OSA based on caregiver-report or medical record reviews. There were no differences in age, sex distribution, APOE-ε4 carrier distribution, or clinical AD diagnostic group by OSA status (Table 1). Those with OSA had greater BMI and were marginally more likely to have Type 2 Diabetes (Table 1). There were no other differences in vascular risk factors or medication usage by OSA status (Table 1). Presence of OSA, OSA duration, or OSA treatment did not differ by clinical AD diagnostic group (Table 2). When OSA was present, duration was reported as being within the past 5 years (*n* = 16) or a lifelong condition (*n* = 25), while treatment was reported as mostly being under control (treated (*n* = 29), inactive (*n* = 5)) with several untreated cases (*n* = 10; Table 2).

**Table 1.** Demographic, diagnostic groups, vascular risk, and medication use by sleep apnea groups.

		Total	No Sleep Apnea	Sleep Apnea	Test statistic, *P*-value
	*N* (%)	116 (100%)	71 (61%)	45 (39%)	-
Demographic	Age [years]	50 ± 8	50 ± 8	50 ± 7	T = 0.46, *p* = 0.65
	Sex [*N* women (%)]	49 (42%)	33 (46%)	16 (36%)	χ ^2^(1) = 1.3, *p* = 0.25
	*APOE ε4* [*N* carriers (%)]	26 (28%)	16 (28%)	10 (29%)	χ ^2^(1) = 0.003, *p* = 0.96
Diagnosis	Cognitively-Stable [*N* (%)]	73 (64%)	49 (70%)	24 (55%)	χ ^2^(3) = 3.8, *p* = 0.29
	MCI-DS [*N* (%)]	26 (23%)	12 (17%)	14 (32%)	
	Possible AD dementia [*N* (%)]	6 (5%)	4 (6%)	2 (5%)	
	Definite AD dementia [*N* (%)]	9 (8%)	5 (7%)	4 (9%)	
Vascular Risk	Hypertension [*N* (%)]	10 (9%)	4 (6%)	6 (13%)	χ ^2^(1) = 2.1, *p* = 0.15
	Hypotension [*N* (%)]	15 (13%)	8 (11%)	7 (16%)	χ ^2^(1) = 0.5, *p* = 0.50
	Blood pressure [mmHg; systolic/diastolic]	109 ± 14/ 65 ± 10	110 ± 13/ 66 ± 10	106 ± 16/ 64 ± 10	F(1) = 2.0, *p* = 0.16/ F(1) = 2.4, *p* = .13
	Type I Diabetes [*N* (%)]	2 (2%)	1 (1%)	1 (3%)	χ ^2^(1) = 0.2, *p* = 0.68
	Type 2 Diabetes [*N* (%)]	7 (6%)	2 (3%)	5 (12%)	χ ^2^(1) = 3.5, *p* = 0.06
	High cholesterol [*N* (%)]	51 (44%)	31 (44%)	20 (44%)	χ ^2^(1) = 0.007, *p* = 0.93
	Body Mass Index [kg/m^2^]	31 ± 7	30 ± 7	32 ± 6	F(1) = 4.6, *p*= 0.03
	Congenital Heart Disease [*N* (%)]	23 (30%)	14 (33%)	9 (27%)	χ ^2^(1) = 0.2, *p* = 0.62
	Heart conditions [*N* (%)]	77 (66%)	44 (62%)	33 (73%)	χ ^2^(1) = 1.6, *p* = 0.21
Other Conditions	Hyperthyroidism [*N* (%)]	4 (3%)	2 (3%)	2 (4%)	χ ^2^(1) = 0.2, *p* = 0.64
	Hypothyroidism [*N* (%)]	73 (63%)	48 (68%)	25 (56%)	χ ^2^(1) = 1.7, *p* = 0.19
	Syncope [*N* (%)]	18 (23%)	9 (20%)	9 (27%)	χ ^2^(1) = 0.5, *p* = 0.48
Medications	Statins [*N* (%)]	39 (34%)	23 (32%)	16 (36%)	χ ^2^(1) = 0.1, *p* = 0.73
	Acetylcholine Esterase Inhibitors [*N* (%)]	17 (15%)	10 (14%)	7 (16%)	χ ^2^(1) = 0.05, *p* = 0.83
	Anti-anxiety [*N* (%)]	8 (7%)	4 (6%)	4 (9%)	χ ^2^(1) = 0.27, *p* = 0.60
	Anti-depressants [*N* (%)]	30 (28%)	16 (25%)	14 (31%)	χ ^2^(1) = 0.50, *p* = 0.48
	Anti-epileptic [*N* (%)]	11 (10%)	6 (9%)	5 (11%)	χ ^2^(1) = 0.09, *p* = 0.77
	Anti-psychotic [*N* (%)]	25 (23%)	16 (25%)	9 (20%)	χ ^2^(1) = 0.37, *p* = 0.54
	Hormone Replacement Therapy [*N* (%)]	1 (1%)	1 (2%)	0 (0%)	χ ^2^(1) = 0.71, *p* = 0.40
	Mood stabilizers [*N* (%)]	11 (10%)	6 (9%)	5 (11%)	χ ^2^(1) = 0.09, *p* = 0.77
	Proton Pump Inhibitors [*N* (%)]	22 (20%)	13 (20%)	9 (20%)	χ ^2^(1) = 0.002, *p* = 0.97
	Thyroid Replacement Medications [*N* (%)]	65 (60%)	40 (63%)	25 (56%)	χ ^2^(1) = 0.53, *p* = 0.47

Note: The numbers of study participants with valid data differed by variable. APOE, *N* = 92; CHD, *N* = 76; Hyperlipidemia, *N* = 76; Type I Diabetes, *N* = 108; Type 2 Diabetes, *N* = 113; BMI = 114; Syncope, *N* = 77; Anti-anxiety through Thyroid Replacement Medications, *N* = 109.

**Table 2.** Sleep apnea duration and treatment status by diagnostic groups. Note that the numbers of study participants with valid data for duration and treatment status were smaller than for self-reported sleep apnea (yes/no). Sleep apnea duration, *N* = 129; Sleep apnea treatment status, *N* = 114.

		Total	Cognitively -Stable	MCI-DS	Probable AD dementia	Definite AD dementia	Test statistic, P-value
Sleep apnea Duration	Not present	88	63	14	3	8	χ ^2^(6) = 9.2, *p* = 0.16
	Lifelong condition	25	13	7	3	2	
	Past 5 years	16	9	6	0	1	
	Past year	0	0	0	0	0	
Sleep apnea Treatment	Not present	70	49	12	4	5	χ ^2^(9) = 13.5, *p* = 0.14
	Inactive	5	4	0	1	0	
	Active, treated	29	16	11	0	2	
	Active, untreated	10	4	3	1	2	

**Figure 1.**
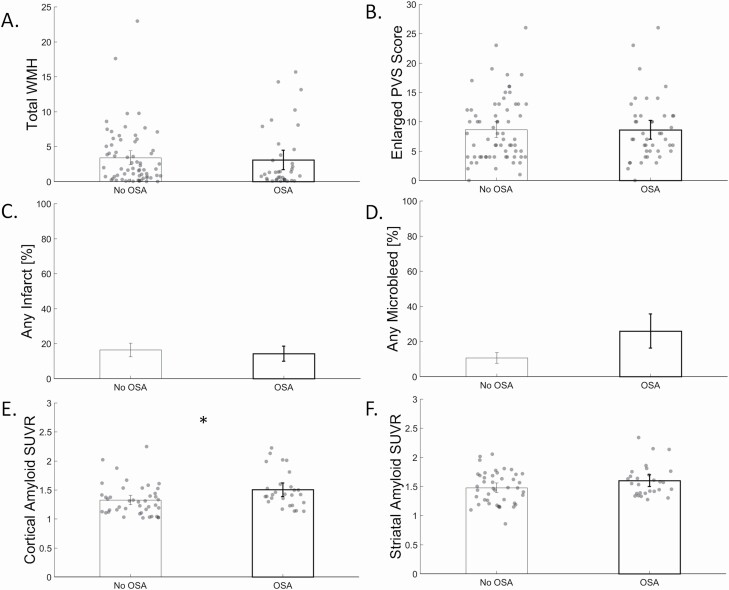
Differences in total white matter hyperintensity (WMH) volume, enlarged perivascular space score, presence of infarcts, presence of microbleeds, cortical amyloid standard uptake value ratio (SUVR), and striatal amyloid SUVR by obstructive sleep apnea (OSA; no OSA = left bars, OSA = right, bolded bars). * indicates *p* < 0.05 for the main effect of OSA.

**Figure 2.**
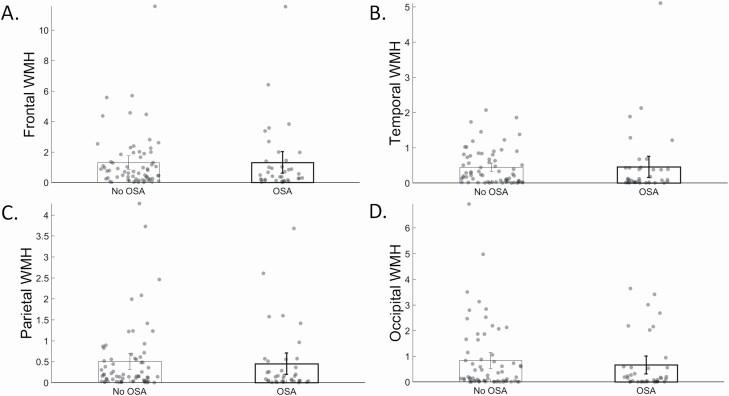
Regional white matter hyperintensity (WMH) volume by obstructive sleep apnea (OSA; no OSA = left bars, OSA = right, bolded bars). * indicates *p* < 0.05 for the main effect of OSA.

Biomarker differences by OSA are shown in Figure 1, while *post hoc* lobar WMH differences by OSA are show in Figure 2. Those with OSA did not differ from those without OSA in total WMH volume (0.33 [-1.18, 1.85], *p* = 0.66, Figure 1, A; frontal (0.19 [-0.60, 0.97], *p* = 0.64, Figure 2, A), temporal (0.12 [-0.14, 0.38], *p* = 0.37, Figure 2, B), parietal (0.04 [-0.26, 0.34], *p* = 0.79, Figure 2, C), and occipital WMH volume (0.02 [-0.43, 0.48], *p* = 0.93, Figure 2, D)), enlarged PVS score (0.10 [-2.1, 2.3], *p* = 0.93, Figure 1, B), presence of microbleeds (3.0 [0.80, 11.1], *p* = 0.10, Figure 1, C), or presence of infarcts (0.80 [0.27, 2.4], *p* = 0.69, Figure 1, D). Those with OSA had greater cortical amyloid burden (0.16 [0.02, 0.31], *p* = 0.03, Figure 1, E), but similar striatal amyloid burden (0.10 [-0.04, 0.23], *p* = 0.18, Figure 1, F), compared with those without OSA; however, the association between OSA and amyloid burden was not apparent after partial volume correction (cortex: 0.16 [-0.04, 0.36], *p* = 0.12; striatum: 0.09 [-0.07, 0.26], *p* = 0.26). There was no association of BMI with any biomarker, but adjustment for BMI in the previous models showed that those with OSA were more likely to have microbleeds than those without OSA (4.5 [1.03, 19.6], *p* = 0.046). There was no association of Type 2 Diabetes with any biomarker, but adjustment for Type 2 Diabetes in the previous models showed that those with OSA were more likely to have microbleeds than those without OSA (3.9 [0.997, 15.5], *p* = 0.050).

**Figure 3.**
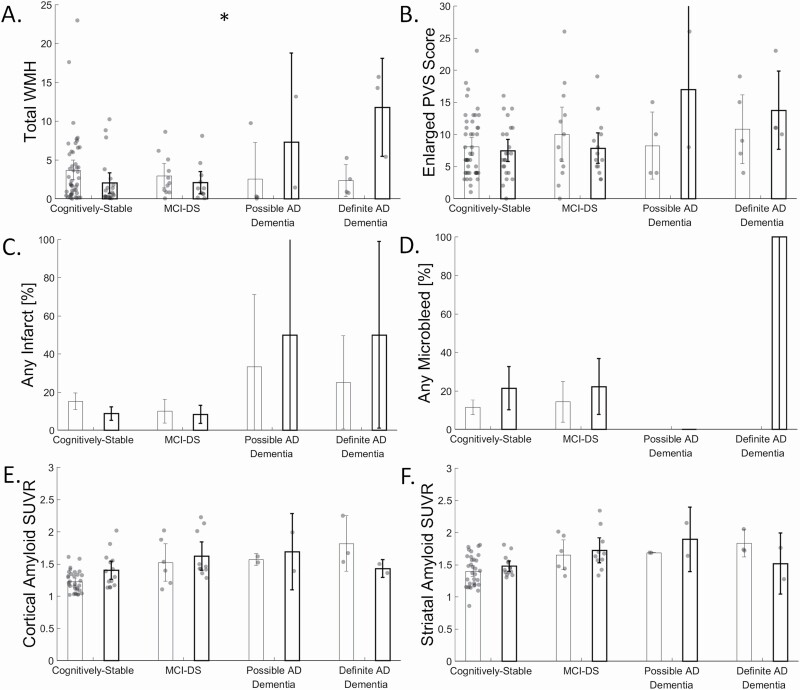
Differences in total white matter hyperintensity (WMH) volume, enlarged perivascular space score, presence of infarcts, presence of microbleeds, cortical amyloid standard uptake value ratio (SUVR), and striatal amyloid SUVR by obstructive sleep apnea (OSA; no OSA = left bars, OSA = right, bolded bars), clinical AD diagnostic group, and their interaction. * indicates *p* < 0.05 for the interaction.

**Figure 4.**
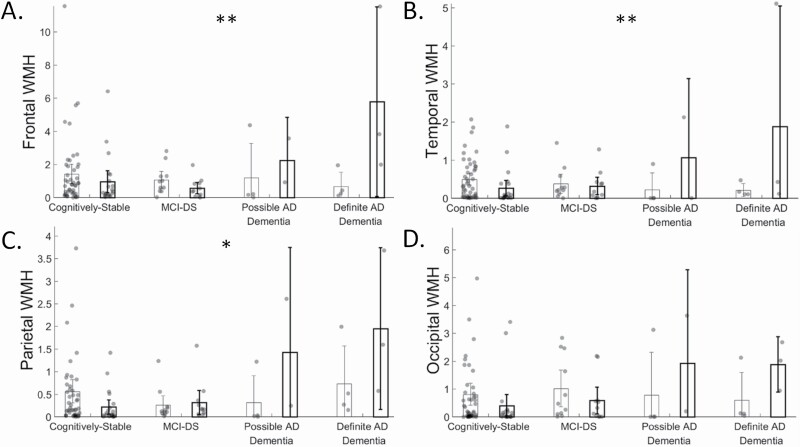
Regional white matter hyperintensity (WMH) volume by obstructive sleep apnea (OSA; no OSA = left bars, OSA = right, bolded bars), clinical AD diagnostic group, and their interaction. * indicates *p* < 0.05 for the interaction. ** indicates *p* < 0.01 for the interaction.

The interaction between OSA and clinical AD diagnostic groups on biomarker level are shown in Figure 3, while the interaction on *post hoc* lobar WMH volumes are shown in Figure 4. We observed monotonically greater total WMH volume (2.3 [0.30, 4.3], *p* = 0.03; frontal (1.1 [0.03, 2.1], *p* = 0.04), temporal (0.45 [0.11, 0.80], *p* = 0.01), and parietal (0.60 [0.20, 1.0], *p* = 0.004), but not occipital WMH volume (0.22 [-0.42, 0.86], *p* = 0.49)), across clinical AD diagnostic groups, as previously reported [30]. We additionally observed an interaction between OSA and clinical AD diagnostic groups in total WMH volume (*F*(3) = 5.0, *p* = 0.003, Figure 3, A; frontal (F(3) = 5.0, *p* = 0.003, Figure 4, A), temporal (*F*(3) = 4.8, *p* = 0.004, Figure 4, B), parietal (*F*(3) = 3.5, *p* = 0.02, Figure 4, C), but not occipital WMH volume (*F*(3) = 1.1, *p* = 0.35, Figure 4, D)), such that the presence of OSA was associated with even greater WMH burden across clinical AD diagnostic groups. Enlarged PVS were monotonically greater across clinical AD diagnostic groups (3.1 [0.08, 6.03], *p* = 0.04), but not differentially in those with and without OSA (*F*(3) = 1.8, *p* = 0.16; Figure 3, B). Infarcts were not more likely across clinical AD diagnostic groups (1.01 [0.47, 2.14], *p* = 0.99), and were not differentially more likely across clinical AD diagnostic groups in those with or without OSA (W(1)=1.6, *p* = 0.21; Figure 3, C). Microbleeds were not more likely across clinical AD diagnostic groups (0.41 [0.06, 2.71], *p* = 0.41), and were not differentially more likely across clinical AD diagnostic groups in those with or without OSA (W(1) = 1.04, *p* = 0.31; Figure 3, D). Cortical amyloid was monotonically greater across clinical AD diagnostic groups (0.19 [0.002, 0.37], *p* = 0.04; with PVC: 0.29 [0.05, 0.53], *p* = 0.02), but was not differentially affected in those with and without OSA (*F*(3) = 2.4, *p* = 0.12, Figure 3, E; with PVC: *F*(3) = 1.6, *p* = 0.19;). Similarly, striatal amyloid was monotonically greater across clinical AD diagnostic groups (0.19 [0.01, 0.37], *p* = 0.04; with PVC: 0.27 [0.06, 0.48], *p* = 0.01), but not differentially in those with and without OSA (*F*(3) = 1.2, *p* = 0.32, Figure 3, F; with PVC: *F*(3) = 0.38, *p* = 0.77). Furthermore, adjustment for BMI showed no differences in results. Furthermore, adjustment for Type 2 Diabetes showed that enlarged PVS were not monotonically greater across clinical AD diagnostic groups (2.9 [-0.16, 5.9], *p* = 0.06).

## Discussion

Among this cohort of older adults with DS, reported OSA was common (39%), was associated with greater cortical amyloid, and interacted with clinical AD diagnosis on ischemic small vessel disease (i.e., WMH). These observations are consistent with previous reports in late-onset AD and raise the possibility that sleep disturbances in adults with DS may contribute to the risk for AD in this population, possibly by increasing the degree to which WMH develops in early clinical AD diagnostic groups, conferring risk for later clinical AD diagnostic groups [32], and/or reducing the time in sleep states when clearance mechanisms are operative [45]. The greater enlarged PVS scores and amyloid burden across clinical AD diagnostic groups were not modified by the presence of OSA, suggesting that they are related to AD and not secondary to OSA. The interaction between OSA and clinical AD diagnosis on WMH volume suggests that the intermittent hypoxic events could interact with AD progression, which itself was associated with greater WMH volume, to amplify cerebrovascular burden for adults with DS. Although the current study presents cross-sectional findings, the results suggest that treating and monitoring treatment compliance for OSA, and its risk factors (i.e. BMI), could reduce the risk of cerebrovascular outcomes during AD progression, improving the quality of life and disease management options for adults with DS; however, OSA treatment may not fully prevent the component of cerebrovascular disease that was associated with clinical AD diagnosis. Thus, cerebrovascular disease biomarkers should be incorporated into the characterization of AD in adults with DS [3,4,46].

As reported previously [30], cerebrovascular disease, as measured by WMH and enlarged PVS, was greater across clinical AD diagnostic groups even after adjusting for OSA suggesting that there is a component of AD-related cerebrovascular disease regardless of factors like OSA. While the intermittent hypoxic events during OSA may not be strong enough to manifest as WMH, they exacerbate WMH when they arise from other causes, such as AD. The greater WMH volumes suggest ischemic small vessel disease across clinical AD diagnostic groups that were even greater for those with OSA compared to those without OSA. High total WMH volumes in those that were Cognitively-Stable without OSA may be influencing the interaction such that the increase across clinical AD diagnostic groups appeared lower in those without OSA compared to those with OSA. However, we have confidence that we are observing a greater increase in WMH volume across clinical AD diagnostic groups from the *posthoc* analyses that demonstrate the expected regional WMH effects along the AD continuum based on previous reports in autosomal dominant AD [31] and late-onset AD [32]. Descriptively, the effect size and confidence intervals for the association between the clinical AD diagnostic group and parietal WMH were furthermore from the null compared to that for frontal and temporal WMH. In contrast, the test statistics for the interaction between OSA and the clinical AD diagnostic group for frontal and temporal WMH were greater compared to that in the parietal WMH. The greater enlarged PVS scores suggest a toxin clearance disturbance across clinical AD diagnostic groups that were unaffected by OSA. Therefore, clearance mechanisms may not be altered by OSA but used less due to fragmented sleep. The likelihood of infarcts did not increase with age, OSA, or clinical AD diagnosis. Previous results suggest that adults with DS are at an increased risk for infarcts at all ages [47], and future work should examine specific factors that increase the risk for infarcts in people with Down syndrome. The lack of an association between microbleeds (which are related to CAA), OSA, and clinical AD diagnosis may be due to the greater likelihood of motion during shorter MRI sequences, resulting in a smaller sample size.

OSA was associated with greater BMI, potentially due to increased narrowing of the upper airway, and marginally associated with Type 2 Diabetes, which may be secondary to greater BMI or a distinct metabolic alteration. OSA was not associated with any biomarkers of cerebrovascular disease in main effect models, except for microbleeds after furthermore adjustment for BMI and Type 2 Diabetes, which may suggest a similar mechanism underlying greater cortical amyloid and vascular amyloid (CAA). Furthermore adjustments for Type 2 Diabetes in interaction models attenuated the AD-related increase in enlarged PVS. These sensitivity analyses suggest that altered metabolic pathways, in addition to chronic intermittent hypoxia and reduced time for toxin clearance through interrupted sleep that are thought to underlie OSA diagnoses, may contribute to associations among OSA, cerebrovascular disease, and AD.

OSA was associated with a higher level of cortical amyloid, but not striatal amyloid, potentially due to reduced time in sleep states when clearance mechanisms are operative. In a cohort of younger adults with DS (38 ± 8 years old), the length of nighttime awakenings was modestly associated with greater amyloid in the striatum, but not the cortex [5]. While people with DS demonstrate increased slow-wave sleep compared with people without DS, this sleep architecture was independent of OSA and slow-wave sleep may be interrupted by the high prevalence of nighttime awakenings [5,48] and greater wake time after sleep onset [49] that occur with OSA. Therefore, OSA and other sleep disturbances may be related to the early amyloid accumulation in the striatum in younger, cognitively-stable adults with DS, but related to the late amyloid accumulation in the cortex in older, cognitively impaired adults with DS, which aligns with previous work showing that amyloid accumulates in the striatum before the cortex in adults with DS [43,44]. While OSA was not associated with cortical amyloid after partial volume correction, the effect may still be biologically meaningful. Partial volume correction may have added additional noise [50], particularly in older adults with DS who tend to move during scanning procedures, which was reflected in the similar effect size, but larger confidence intervals. Previous work in adults without DS showed greater amyloid in the posterior cingulate gyrus and temporal cortex in those with OSA, potentially related to clearance mechanisms [22], which differed by AD disease stage [26,51–53]. Together, these findings support future studies in larger cohorts across the lifespan in adults with DS with direct measurements of sleep quality and disturbances that will help elucidate the mechanism (e.g. time spent in slow-wave sleep) between OSA and amyloid burden.

Subjectively, it appeared that numerically the relationship of OSA on cerebrovascular and amyloid biomarkers were largest between those with no OSA and those with active, untreated OSA or those with lifelong OSA, but sample sizes were too small to draw inferential conclusions from this preliminary observation. In previous studies, following 3 months of continuous positive airway pressure (CPAP) treatment, adults without DS and with OSA showed improvements in memory, attention, and executive function performance [20]. Post-treatment improvements in cognition were associated with improvements in white matter microstructure measured through DTI [21], but no work was done on WMH or other image-based cerebrovascular biomarkers. Separate evidence suggests that WMH affect white matter microstructure [54], perhaps linking CPAP treatment to lower WMH and improved white matter microstructure. Importantly, CPAP may be a viable option in adults with DS with data showing better adherence rates (35% [11]) compared to the non-DS population (18% [55]). A study in the Alzheimer’s Disease Neuroimaging Initiative (ADNI) showed that OSA was associated with an earlier age of onset for MCI and AD in adults without DS and that CPAP use delayed the age of onset for MCI [15], suggesting that OSA may be a modifiable target for AD progression.

The results support future investigations of the links between OSA, cerebrovascular disease, and amyloid in adults with DS across the AD continuum. In this study, the diagnosis of OSA was ascertained via self- or informant-report and/or by review of available medical records and not diagnosed prospectively or assessed with polysomnography, actigraphy, or oxygen desaturation index. Previous studies suggest that OSA is often undiagnosed in adults with DS and the prevalence based on objective measures may be greater than 78% [11,12]. Thus, it is likely that there were individuals included in the study with undiagnosed OSA [56]. However, the inclusion of such undiagnosed cases would attenuate associations and bias towards the null hypothesis. Preliminary data suggest that implementing actigraphy or WatchPAT 300 for OSA measurement might be feasible in adults with DS [5,6,12,57]. The prevalence of OSA increases with age, which is tied to amyloid accumulation and AD dementia, in people with DS [48]. Participants in the ADDS study are all over the age of 40 years; thus, it is possible that the sample may not fully represent the Down syndrome population. Furthermore, the sample was restricted to those who completed MRI and PET imaging with sufficient quality (e.g. not too much motion) and therefore may not accurately represent adults with DS who were unable to complete imaging. Missingness for each biomarker was not associated with age, sex, the presence of an APOE-ε4 allele, clinical AD diagnosis, or OSA, except missingness in microbleed and increasing age. The multiple biomarker approach provides a comprehensive understanding of the associations between cerebrovascular disease, amyloid, OSA, and clinical AD diagnostic groups, but future work with larger sample sizes will be needed to confirm the findings and to consider OSA duration and treatment. The cross-sectional nature of this analysis in older adults with DS cannot determine time-ordered nature of associations, and it is possible that greater amyloid and cerebrovascular burden may contribute to OSA. There is also evidence that OSA affects young and middle-aged adults differently than older adults [27]. The Alzheimer’s Biomarker Consortium-Down Syndrome (ABC-DS) study [58], which combines a cohort of young and middle-aged adults with DS (Neurodegeneration in Aging Down Syndrome; NiAD) and a cohort of older adults with DS (ADDS), will be collecting longitudinal data, including self-reported and measured (i.e. actigraphy) OSA, that may be suitable to address these issues.

Overall, OSA is common among adults with DS, associated with BMI and cortical amyloid burden, and associated with even greater small vessel cerebrovascular disease across clinical AD diagnostic groups. Fully compliant OSA treatment may not prevent the development of cerebrovascular disease and amyloid in adults with DS across the AD continuum, but may attenuate their development. The findings have implications for AD management strategies in adults with DS by suggesting that efforts to monitor and effectively treat OSA, among other factors [59,60], could be key to optimal brain aging.

## Data Availability

Data from the ADDS study are available upon request from the Image Data Archive at the Laboratory of Neuro Imaging (LONI).
